# Evolution and diversity of community-associated methicillin-resistant *Staphylococcus aureus *in a geographical region

**DOI:** 10.1186/1471-2180-11-215

**Published:** 2011-09-29

**Authors:** Geoffrey W Coombs, Stefan Monecke, Julie C Pearson, Hui-leen Tan, Yi-Kong Chew, Lynne Wilson, Ralf Ehricht, Frances G O'Brien, Keryn J Christiansen

**Affiliations:** 1 Australian Collaborating Centre for Enterococcus and Staphylococcus Species (ACCESS) Typing and Research. PathWest Laboratory Medicine - WA, Royal Perth Hospital, Wellington Street, Perth, Western Australia, 6000 Australia; 2 School of Biomedical Sciences. Curtin University of Technology, GPO Box U1987, Perth, Western Australia, 6000 Australia; 3 Alere Technologies GmbH, Löbstedter Straße 103-105, D-07749 Jena, Germany

## Abstract

**Background:**

Community-associated methicillin-resistant *Staphylococcus aureus *(CA-MRSA) was first reported in remote regions of Western Australia and is now the predominant MRSA isolated in the state. The objective of this study is to determine the genetic relatedness of Western Australian CA-MRSA clones within different multilocus sequence type (MLST) clonal clusters providing an insight into the frequency of *S. aureus *SCC*mec *acquisition within a region.

**Results:**

The CA-MRSA population in Western Australia is genetically diverse consisting of 83 unique pulsed-field gel electrophoresis strains from which 46 MLSTs have been characterised. Forty five of these sequence types are from 18 MLST clonal clusters and two singletons. While SCC*mec *IV and V are the predominant SCC*mec *elements, SCC*mec *VIII and several novel and composite SCC*mec *elements are present. The emergence of MRSA in diverse *S. aureus *clonal clusters suggests horizontal transmission of the SCC*mec *element has occurred on multiple occasions. Furthermore DNA microarray and *spa *typing suggests horizontal transfer of SCC*mec *elements has also occurred within the same CC. For many single and double locus variant CA-MRSA clones only a few isolates have been detected.

**Conclusions:**

Although multiple CA-MRSA clones have evolved in the Western Australian community only three clones have successfully adapted to the Western Australian community environment. These data suggest the successful evolution of a CA-MRSA clone may not only depend on the mobility of the SCC*mec *element but also on other genetic determinants.

## Background

Based on phenotypic and genotypic typing methods, community onset methicillin-resistant *Staphylococcus aureus *infections are caused by healthcare-associated MRSA (HA-MRSA) strains, which appear to have been transferred from hospitals or healthcare facilities into the community by patients or healthcare workers [1], or by community-associated MRSA (CA-MRSA) strains, which have been isolated from people who have had little or no contact with healthcare facilities or healthcare workers [2]. This distinction between community and healthcare facility however has become blurred with the replacement of HA-MRSA with CA-MRSA in hospitals [3,4].

In contrast to HA-MRSA, CA-MRSA strains are generally more susceptible to non beta-lactam antibiotics, grow significantly faster, have different clonal backgrounds, carry smaller staphylococcal cassette chromosome *mec *(SCC*mec*) elements (most commonly SCC*mec *type IV or type V), have enhanced virulence properties and frequently harbor genes expressing Panton-Valentine leukocidin (PVL) [5-8]. Rather than a worldwide spread of a single clone multiple CA-MRSA clones have emerged from diverse genetic backgrounds. Several well characterized CA-MRSA clones predominate in different regions: Sequence type (ST) 8-IV [2B] (USA300) and ST1-IV [2B] (USA400) in North America [9,10]; ST80-IV [2B] (European clone) in Europe [8], North Africa [11] and the Middle East [12]; ST59-V [5C2&5] (Taiwan clone) in Taiwan [13]; ST93-IV [2B] (Queensland clone) in Australia [14], ST30-IV [2B] (South West Pacific [SWP] CA-MRSA) in the Western Pacific [15,16], and ST772-V [5C2] (Bengal Bay clone) in India and Bangladesh [17]. Transmission of these clones into other regions has occurred [18,19]. This occurrence of concurrent epidemics of CA-MRSA in many countries by different clones has been striking. Equally noteworthy are a number of common features of these epidemics, prominent among them the ability to cause severe infections in young otherwise healthy people and the carriage of the *lukF-PV/lukS-PV *PVL encoding genes by the organism.

The earliest report of CA-MRSA infections involved indigenous people living in remote communities in the sparsely populated Kimberley region of Western Australia (WA) [20]. Approximately 50% of the people in this region are indigenous, many of whom live in poor socioeconomic conditions. Infected skin lesions and staphylococcal sepsis occur frequently and empirical antistaphylococcal therapy is often prescribed. Colloquially known as "WA-MRSA", the early isolates have a similar pulsed-field gel electrophoresis (PFGE) pattern and have subsequently been characterized as a single clone; PVL-negative WA5 (ST8-IV/*spa *t008) [21]. By 2006 22 CA-MRSA clones were identified in WA, with PVL-negative WA 1 (ST1-IV [2B]/t127) replacing WA5 as the predominant clone [22]. At this time CA-MRSA from indigenous people living in remote areas outside of WA were reported in the Northern Territory [23], Queensland [24] and Central Australia [25]. As may be expected in a geographically large country with relatively few dense concentrations of population, often separated by large areas of desert, different CA-MRSA clones evolved in these communities.

In 1982 colonization or infection with MRSA became a notifiable condition in WA. For infection control purposes all MRSA isolated in the state since 1997 have been referred to the Australian Collaborating Centre for *Enterococcu*s and *Staphylococcus Species *(ACCESS) Typing and Research where based on molecular markers they are characterized as either HA-MRSA or CA-MRSA [26]. Although a state-wide policy of screening all patients and healthcare workers who have lived outside the state for MRSA has prevented HA-MRSA from becoming endemic in Western Australian hospitals, it has not prevented CA-MRSA from becoming established in the community. In WA the public health system is divided into two metropolitan health regions and seven country health regions. The state encompasses an area of 1.02 million square miles and has a population of approximately 2.24 million people. In 1983, the overall rate of MRSA notifications was 10 per 100,000 persons in the rural country health regions and 7/100,000 in the metropolitan regions [27]. By 2006 notifications rates throughout the state had increased to 179/100,000 persons of which 144/100,000 were CA-MRSA. In the metropolitan health regions the CA-MRSA notification rate was 134/100,000 whilst in the Kimberley health region the CA-MRSA notification rate had increased 40-fold to 391/100,000 [18].

CA-MRSA is thought to emerge when a locally prevalent strain of methicillin susceptible *S. aureus *(MSSA) acquires a SCC*mec *element and utilizes mobile genetic elements and single nucleotide polymorphisms to establish local and geographic niches [28]. As WA is a remote region in which all MRSA isolates are referred to a central typing laboratory it is an ideal environment to study the emergence and evolution of CA-MRSA. MLST, SCC*mec*, *spa *typing and microarray DNA is performed on all isolates with a unique PFGE pulsotype. The aim of this study is to determine the genetic relatedness of WA CA-MRSA clones within different MLST clonal clusters (CC) providing an insight into the frequency of *S. aureus *SCC*mec *acquisition within a region. The genetic profile of these clones may also offer an explanation why only a few WA CA-MRSA clones have successfully adapted to the community environment.

## Results

The 83 unique PFGE strains isolated in Western Australia from 1989 to 2010 were *nuc *and *mecA *gene positive by PCR. The DNA microarray *S. aureus *species markers *gapA *(glyceraldehyde 3-phosphate dehydrogenase) and *rrn STAU *(*S. aureus *ribosomal marker) were detected in all strains. The array's linear primer elongation method detected the *katA *(catalase A), *coA *(coagulase), *nuc*, *spa *(protein A) and *sbi *(IgG-binding protein) *S. aureus *species markers in 78 strains. These markers were either not detected or detected only by random amplification in five strains (WA8, WA47, WA72, WA76 and WA79).

**Figure 1:**
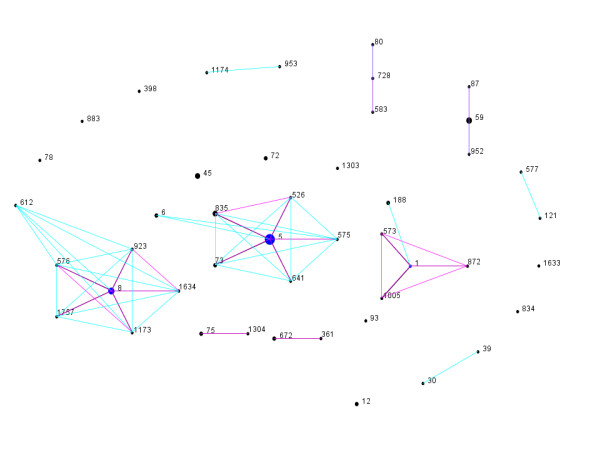
**eBURST generated population snapshot of CA-MRSA clones isolated in Western Australia (http://www.mlst.net/)**. Each sequence types (STs) is represented by a black dot. The ancestral ST of a clonal complex is represented by a blue dot. The size of the dot reflects the number of WA CA-MRSA clones with this ST. STs that diverge at no more than one of the seven MLST loci belong to the same clonal complex. Double locus variants (DLVs) are included if the linking single locus variant (SLV) was present in the MLST database. SLVs and DLVs of a sequence type are represented by pink and blue line respectively. Purple lines represent overlapping pink and blue lines.

Forty six STs were identified by MLST. Using the MLST website's eBURST V3 algorithm 45 STs were grouped into 18 CCs and two singletons (Figure 1). The CC for WA76 (ST1303) has not been determined.

Several SCC*mec *types and subtypes, novel SCC*mec*s, and composite SCC*mec*s were identified. Forty five strains harbor SCC*mec *IVa-d [2B] (31 IVa, 2 IVb, 9 IVc, 3 IVd), 12 strains SCC*mec *V [5C2] and two strains SCC*mec *VIII [4A]. Two strains have non typeable SCC*mec *IV subtypes and four strains have a SCC*mec *element with a novel *ccr *gene complex including three with a class B *mec *gene complex and one with a class A *mec *complex. Eighteen strains harbor SCC*mec *elements with composite *ccr *gene complexes including 12 with SCC*mec *V [5C2&5] (5C2 plus *ccrC1 *allele 8), three with SCC*mec *IVa [2B]&5 (2B plus a type 5 *ccr *gene complex), one with V (5C2)&2 (5C2 plus a type 2 *ccr *gene complex) and two with V [5C2&5]&2 (a composite SCC*mec *V element plus a type 2 *ccr *gene complex).

The MLST, *spa *type, *agr *type, capsule type, SCC*mec*, antibiogram, resistance genotype, lukF/S-PVL genes, enterotoxin genes and bacteriophage associated virulence genes of each unique PFGE strain are provided in Additional File 1. Information on target genes, probes, and primers is provided in Additional File 2. Complete hybridization profiles for the individual strains can be provided on request.

### Clonal Complex 1

CC1 contains five strains including the PVL positive Bengal Bay clone (ST772 [a single locus variant {slv} of ST1]-V [5C2]/t3387). This strain is epidemiologically linked to a healthcare worker from India and is not considered a WA CA-MRSA.

Based on the *agr*/capsule and SCC*mec *type, the remaining four strains are divided into two groups:

#### Group 1

*agr *type III/capsule type 8 SCC*mec *IVa [2B] contains PVL negative WA1 (ST1/t127), WA45 (ST872 [slv of ST1]/t127), and WA57 (ST1005 [ST1 slv]/t127). WA1 and WA45 harbor a *ccrA-1 *and *ccB-1 *gene complex and Q6GD50 (fusidic acid resistance marker) indicating the presence of the mobile fusidic acid SCC element SCC*fur*. WA1 is known to carry multiple plasmids such as a 2-kb plasmid encoding resistance to erythromycin [29] and this presumably accounts for the differences in the antibiogram and resistance genotype for WA1, WA45 and WA57. In addition to enterotoxin genes the three strains harbor a type D immune evasion cluster [IEC] (*seA*+*sak*+*scn*) [30]. Group 2

*agr *type II/capsule type 5 SCC*mec *V [5C2] contains PVL negative WA10 (ST573 [ST1 slv]/t5073. WA10 carries several enterotoxin genes including the enterotoxin *egc *cluster [*seG*+*seI*+*seM*+*seN*+*seO*+s*eU*/*Y*]). Unlike WA1, WA45 and WA57, WA10 does not carry the type D IEC, the pathogenicity island harboring the leukocidin D/E component, the protease *splA *gene and the *hsdS *gene. The *ssl*/*set *genes and cell surface adhesions encoding genes of WA10 are closely related to the Bengal Bay clone.

### Clonal Complex 5

CC5 contains 27 strains. Based on the *agr/*capsule type the isolates are divided into two groups which are further divided into subgroups based on the SCC*mec *type.

#### Group 1

*agr *type I/capsule type 8 (2 strains)

i. SCC*mec *IVa [2B] contains WA51 (ST6 [ST5 dlv]). The protein A variable region in WA51 could not be amplified and therefore a *spa *type cannot be allocated.

ii. SCC*mec *IVa [2B]&5 contains WA66 (ST6/t701).

WA51 and WA66 harbor a type D IEC Neither strain harbors the *lukF-PV/lukS-PV *PVL encoding genes.

#### Group 2

*agr *type II/capsule type 5 (25 strains)

Unlike Group 1 strains, these 25 strains harbour the enterotoxin *egc *cluster. Ten *spa *types were identified, of which nine are closely related: t002, t045, t071, t442, t688, t1265, t2666, t3378, t4065.

i. SCC*mec *IVa [2B] contains WA3 (ST5/t002), WA64 (ST5/t3778), WA71 (ST5/t002), WA82 (ST5/t002), WA25 (ST575 [ST5slv]/t002), WA50 (ST73 [ST5slv]/t002) and WA65 (ST73/t002). PVL negative WA3, WA71, WA82, WA25, WA50 and WA65 harbor a type F IEC (*seP*+*sak*+*chp*+*scn*). PVL positive WA64 harbors a type A IEC (*seA*+*sak*+*chp*+*scn*). WA64 and WA65 also harbor *edinA *(epidermal cell differentiation inhibitor A gene).

ii. SCC*mec *IVc [2B] contains PVL negative WA74 (ST5/t002) which harbors a type F IEC.

iii. SCC*mec *IV [2B] contains PVL negative WA39 (ST526 [ST5slv]/t4065) which has a non typeable SCC*mec *IV [2B] element and a type B IEC (*sak*+*chp*+*scn*).

iv. SCC*mec *V [5C2] contains PVL negative WA14 (ST5/t442), WA35 (ST5/t688), WA81 (ST5/t045) [a non related spa type] and WA90 (ST5/t1265). WA81 harbors a type F IEC; WA14 and WA90 a type G IEC (*seP*+*sek*+*scn*) and WA35 a type B IEC.

v. SCC*mec *V [5C2&5] contains PVL negative WA11 (ST5/t045), WA86 (ST5/t002), WA34 (ST5/t458), WA80 (ST5/t071), WA85 (ST5/t2666), and WA87 (ST835 [ST5slv]/t002). WA85 and WA86 harbor a type F IEC; WA34, WA80 and WA87 a type B IEC and WA11 a type E IEC (*sak *+ *scn*). WA80 harbors the ACME (arginine catabolic mobile element) genes.

vi. SCC*mec *V [5C2]&2 contains PVL negative WA61 (ST641 [ST5slv]/t002) which harbors a type E IEC.

vii. SCC*mec *V [5C2&5]&2 contains PVL negative WA40 (ST835 [ST5slv]/t002) and WA46 (ST835/t002). WA40 harbors a type B IEC while WA46 a type E IEC.

viii. SCC*mec *novel [novel B] contains PVL negative WA18 (ST5/t002), WA21 (ST5/t002) and WA48 (ST835/t002) harboring *ccrA-1 *and a class B *mec *complex (*mecA *and a truncated *mecR1 *genes). WA18 harbors a type F IEC; WA21 a type D IEC; and WA48 a type B IEC.

### Clonal Complex 8

The 12 CC8 strains are all *agr *type I/capsule type 5. Seven closely related *spa *types were identified: t008, t024, t064, t334, t711, t1635, t2238.

The CC8 strains include the ST8-MRSA-IVc [2B]/t008 USA300 MRSA clone [31]. Based on the SCC*mec *type the remaining 11 strains are divided into seven subgroups:

i. SCC*mec *IVa [2B] contains WA5 (ST8/t008), WA6 (ST8/t008), WA62 (ST923 [ST8slv]/t1635), and WA83 (ST1634 [ST8slv]/t711). WA5, WA62, and WA83 harbor a type B IEC. An IEC was not detected in WA6. Unlike the other WA CC8 strains, WA62 is PVL positive.

ii. SCC*mec *IVd [2B] contains WA58 (ST1173 [ST8slv]/t064) and WA20 (ST612 [ST8dlv]/t064) which harbor a type D IEC.

iii. SCC*mec *IVa [2B]&5 contains WA92 (ST1757 [ST8slv]/t024) which does not harbor an IEC.

iv. SCC*mec *IV [2B] contains WA31 (ST576 [ST8slv]/t334) which does not harbor an IEC. The SCC*mec *IV element is non typeable.

v. SCC*mec *V [5C2] contains WA77 (ST8/t008) which harbors a type D IEC, the ACME determinant, and SCC*fus*.

vi. SCC*mec *V ([5C2&5]) contains WA53 (ST8/t2238) which harbors a type D IEC.

vii. SCC*mec *VIII (4A) contains WA16 (ST8/t024) which harbors a type D IEC.

### Clonal Complex 12

CC12 contains two *agr *group II/capsule type 8 strains which harbor a type G IEC. Neither strain harbor the *lukF-PV/lukS-PV *PVL encoding genes.

Based on the SCC*mec *type the two strains are divided into two subgroups:

i. SCC*mec *IVa [2B] contains WA69 (ST12/t160).

ii. SCC*mec *novelA contains WA59 (ST12/t160) which harbors a class A *mec *complex (*mecA*, complete *mecR1 *and *mecI *regulatory genes). The *ccr *genes were not detected by DNA microarray and did not amplify with PCR primers.

### Clonal Complex 30

CC30 contains two *agr *group III/capsule type 8 strains: PVL positive ST30-IVc [2B]/t019 and PVL negative WA68 (ST39 [ST30dlv]-IVc [2B]/t2643). Their protease, haemolysin, leukocidin, *ssl*/*set*, *hsdS*, and cell surface adhesion profiles are not homogeneous and their *spa *types are not closely related.

The DNA microarray profile of ST30-IVc [2B]/t019 is homogeneous with the South Western Pacific (SWP) ST30-IV clone as is therefore not considered a WA CA-MRSA.

WA68 harbors a type D IEC and *tst-1*genes.

### Clonal Complex 45

CC45 contains four PVL negative strains. Based on the *agr *group/capsule type the four isolates are divided into two groups which are further divided into subgroups based on the SCC*mec *type.

#### Group 1

*agr *group I/capsule 8 (two strains)

i. SCC*mec *IVa [2B] contains WA75 (ST45/t1424).

ii. SCC*mec *V [5C2] contains WA4 (ST45/t123) which harbors *tst1 *genes.

Both strains harbor a type B IEC. The *spa *types are not closely related.

#### Group 2

*agr *group IV/capsule type 8 (two strains)

i. SCC*mec *IVc [2B] contains WA23 (ST45/t1575)

ii. SCC*mec *V [5C2&5] contains WA84 (ST45/t1081).

Both strains harbor a type B IEC and closely related *spa *types.

### Clonal Complex 59

CC59 *agr *type I/capsule type 8 contains seven strains.

The DNA microarray profiles of ST59/ST952-V [5C2&5] t437/t1950 are homogeneous with the Taiwan clone and therefore are not considered WA CA-MRSA [32].

Based on the SCC*mec *types the remaining five strains are divided into three subgroups:

i. SCC*mec *IVa [2B] contains PVL positive WA55 and WA56 (ST59/t437). WA55 harbors a type B IEC while WA56 a type A IEC.

ii. SCC*mec *IVb [2B] contains two PVL negative strains with unrelated *spa *types: WA73 (ST59/t528) and WA24 (ST87 [ST59slv]/t216). WA73 harbors a type C IEC (*chp*+*scn*) and WA24 a type B IEC.

iii. SCC*mec *IVa [2B]&5 contains PVL negative WA15 (ST59/t976) which harbors a type A IEC.

### Clonal Complex 72

CC72 contains two *agr *group I/capsule type 5 strains with closely related *spa *types. Based on the SCC*mec *type the two strains are divided into two subgroups:

i. SCC*mec *IVa [2B] contains PVL positive WA44 (ST72/t791) harboring a type B IEC.

ii. SCC*mec *V (5C2) contains PVL negative WA91 (ST72/t3092) harboring a type E IEC and *tst1 *genes.

### Clonal Complex 75

CC75 contains three PVL negative strains which are *agr *group/capsule nontypeable by DNA microarray: WA8 (ST75-IVa [2B]), WA79 (ST75-IVa [2B]) and WA72 (ST1304 [ST75slv]-IVa [2B]) [33]. The three strains have the same *spa *sequence (259-23-23-17-17-17-23-23-23-17-16) which has not been allocated a *spa *type number by the Ridom website. The three strains harbor a type E IEC.

### Clonal Complex 80

CC80 contains three PVL positive *agr *group III/capsule type 8 strains: ST80-IVc [2B]/t044, ST583 [ST80slv]-IVc [2B]/t044, and ST728 [ST80slv]-IVc [2B]/t044. The DNA microarray virulence profiles are identical with the European ST80-IV [2B] clone and therefore the three strains are not considered WA CA-MRSA.

### Clonal Complex 97

CC97 contains two PVL negative *agr *group I/capsule type 5 strains with closely related *spa *types: WA54 (ST953[ST97dlv]-IVa [2B]/t359) and WA63 (ST1174[ST97dlv]-IVa [2B]/t267). The strains harbor a type E IEC.

### Clonal Complex 121

CC121 contains two PVL negative *agr *group IV/capsule type 8 strains with closely related *spa *types. The two strains harbor a type E IEC and based on the SCC*mec *type, are divided into two subgroups:

i. SCC*mec *V [5C2] contains WA22 (ST577 [ST121 dlv]/t3025) which harbors *etA *(exfoliative toxin serotype A) and *edinA *genes.

ii. SCC*mec *V [5C2&5] contains WA93 (ST121/t159).

### Clonal Complex 188

CC188 contains two PVL negative *agr *group I/capsule type 8 strains: WA38 and WA78 (ST188-IVa [2B]/t189). The two strains have a type B IEC.

### Clonal Complex 361

CC361 contains three PVL negative *agr *group I/capsule type 8 strains. The *spa *types are closely related. Based on the SCC*mec *type the three strains are divided into three subgroups:

i. SCC*mec *IVa [2B] contains WA29 (ST672 (ST361slv)/t1309) which harbors a type E IEC and *tst1 *genes.

ii. SCC*mec *V [5C2] contains WA70 (ST672/t1309).

iii. SCC*mec *VIII [4A] contains WA28 (ST361/t315) which harbors a type B IEC.

The following CCs contained a single strain:

### Clonal Complex 9

PVL negative WA13 (ST834-IVc [2B]/t3029) is *agr *group I/capsule type 8 and harbors a type B IEC and *tst1 *genes.

### Clonal Complex 88

PVL negative WA2 (ST78-IVa [2B]/t3205) is *agr *group III/capsule type 8 and harbors a type B IEC.

### Clonal Complex 152

PVL positive WA89 (ST1633-V [5C2]/t355) is *agr *group I/capsule type 5 and harbors a type E IEC and *edinB *genes.

### Clonal Complex 398

Although PVL negative ST398-V [5C2&5]/t034 is frequently associated with livestock, the strain is increasingly isolated from human patients [34]. Rarely identified in Australia, the DNA microarray profile of this isolate is homogeneous with the European livestock-associated ST398 strain and is therefore not considered a WA CA-MRSA.

### WA76 (Clonal Complex not Determined)

PVL negative WA76 (ST1303-IVa [2B]) is *agr *group III with a non typeable capsule by DNA microarray. The *spa *sequence (259-25-17-17-16-16-16-16) has not been allocated a *spa *type number by the Ridom website.

### Queensland Clone (Singleton)

PVL positive ST93-IVa [2B]/t202 is *agr *group III/capsule type 8 and harbors a type B IEC. The DNA microarray profile is homogeneous with the Queensland clone. Due to its origin and widespread distribution outside WA the Queensland clone is not considered a WA CA-MRSA.

### WA47 (Singleton)

PVL negative WA47 (ST883-IVd [2B]/t7462) has a non typeable *agr *group/capsule type by DNA microarray.

## Discussion

As all MRSA isolated in WA are referred to a central typing laboratory it is possible to investigate the emergence and evolution of CA-MRSA in a remote region.

**Figure 2:**
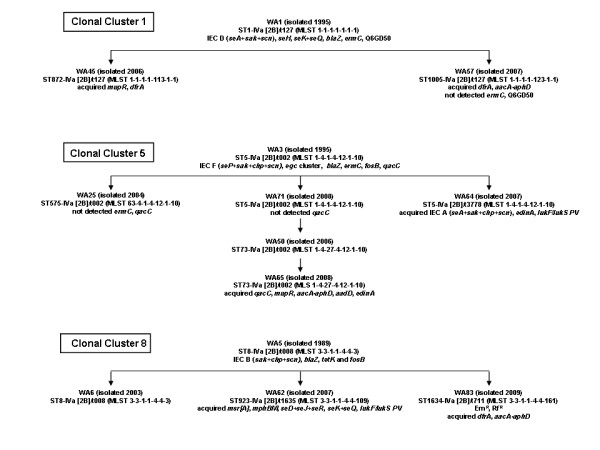
**Proposed evolution of CA-MRSA from WA-1 (ST1-MRSA-IV), WA-3 (ST5-MRSA-IV) and WA-5 (ST8-MRSA-IV)**.

Prior to the global evolution and expansion of CA-MRSA, five CA-MRSA clones were identified in the indigenous population living in the remote communities of the sparsely populated Kimberley, Pilbara and Eastern Goldfield regions of WA [29]. These five PVL negative clones include: WA1 (CC1: ST1-IVa [2B]/t127), WA2 (CC88: ST78-IVa [2B]/t3205), WA3 (CC5: ST5-IVa [2B]/t002), WA4 (CC45 ST45-V (5C2)/t123) and WA5 (CC8: ST8-IVa [2B]/t008). WA5 and WA1 were originally isolated from clinical specimens in 1989 and 1995 respectively, and WA2, WA3 and WA4 from nasal carriage specimens in 1995. The emergence of CA-MRSA clones in different MLST clonal clusters indicates horizontal transmission of the SCC*mec *element into *S. aureus *has occurred on at least five occasions in these remote communities: SCC*mec *IVa [2B] into CC1 (ST1), CC5 (ST5), CC8 (ST8), CC88 (ST78), and SCC*mec *V [5C2] into CC45 (ST45). Based upon the *spa *type and the DNA microarray profile at least six evolutionary events have occurred on at least three occasions from these clones (ie vertical transmission of the SCC*mec *element): twice from WA1, WA3 and WA5 (Figure 2). Vertical transmission of the SCC*mec *element has not been identified for WA4 or WA2.

The emergence of WA1, WA2 and WA3 has been due to the acquisition and insertion of the small and highly mobile type IVa [2B] SCC*mec *element, presumably harbored by methicillin resistant coagulase negative staphylococci (MRCNS). Several hypotheses to explain the transmission of a SCC*mec *element from MRCNS to *S. aureus *have been proposed including the increased use of antimicrobials within a community [35]. Many of the Kimberley indigenous population live in poor socioeconomic conditions. Staphylococcal skin lesions, commonly resulting from scabies infestation, trachoma and venereal diseases such as chlamydia and gonorrhea occur frequently in this population. Consequently empirical therapy using β-lactamase stable penicillins and azithromycin is often prescribed [36]. The frequent use of these antimicrobials may have assisted in the acquisition of the SCC*mec *element and *erm *genes into *S. aureus*. Genetic studies however have shown these newly emerged CA-MRSA clones did not originate in the predominant methicillin-susceptible *S. aureus *(MSSA) clones found in these communities, suggesting not all clones are able to acquire or retain the SCC*mec *element [37]. The subsequent dissemination of WA1, WA2 and WA3 into the wider community suggests the acquisition of the SCC*mec *element and the *erm *genes has given these clones a selective advantage. WA4 and WA5 however have not been successful in spreading beyond the indigenous communities suggesting the acquisition of the SCC*mec *element does not provide a universal selective advantage.

Many of the remaining 46 CA-MRSA clones, identified between July 2003 and June 2010, were not isolated in remote WA indigenous communities. The geographical spread of CA-MRSA over long distances and across cultural borders is believed to be a rare event compared to the frequency in which the SCC*mec *element is acquired by *S. aureus *[38]. Most of these clones are therefore likely to have evolved in WA. Some clones are slvs and dlvs of pre-existing CA-MRSA, and their SCC*mec *type, *spa *type and DNA microarray profile suggests vertical transmission of the SCC*mec *element has occurred. However the emergence of MRSA in several unrelated *S. aureus *clonal clusters suggests horizontal transmission of the SCC*mec *element has also occurred. SCC*mec *typing and *spa *typing and DNA microarray results also suggests horizontal transfer of SCC*mec *elements has occurred into the same CC on more than one occasion.

Although several SCC*mec *elements have been acquired by multiple *S. aureus *clones from which many CA-MRSA clones have emerged, only a few clones have successfully adapted to the WA community environment. Between July 2009 to June 2010 4,691 MRSA were referred to ACCESS Typing and Research of which 3,931 were characterized as CA-MRSA. Overall 84% (3,024) of isolates were from clinical infections and the 16% (907) from colonized patients. Approximately 88% of CA-MRSA were identified as WA1 (40%), WA2 (24%) and WA3 (8%). For most clones, including WA4 and WA5 only a few isolates were detected. (http://www.public.health.wa.gov.au/3/896/3/camrsa.pm).

For many slv and dlv CA-MRSA only a small number of isolates have been detected suggesting changes in the housekeeping genes may have conferred a fitness cost or did not allow the SCC*mec *element to be maintained. For example WA45 and WA57 are slvs of ST1 and their SCC*mec *and *spa *type and DNA microarray profile suggest they have evolved from WA1 (Figure 2). WA45 was first identified in 2006 and WA57 in 2007. Although WA1 has become the most successful CA-MRSA clone in the WA community only one isolate of WA45 and two isolates of WA56 have so far been identified (http://www.public.health.wa.gov.au/3/896/3/camrsa.pm).

Six PVL positive pandemic CA-MRSA clones (plus three closely related clones) have been isolated in WA: Bengal Bay CA-MRSA (ST772-V [5C2]/t3387), USA300 MRSA (ST8-IVc [2B]/t008), SWP CA-MRSA (ST30-IVc [2B]/t019), Taiwan CA-MRSA (ST59-V [5C2&5]/t437 and the slv ST952-V [5C2&5]/t1950), European CA-MRSA (ST80-IVc [2B]/t044 and the slvs, ST583-IVc [2B]/t044 and ST728-IVc [2B]/t044), and the Queensland CA-MRSA (ST93-IVa [2B]/t202). The epidemiology of the USA300 and Taiwan CA-MRSA clones in WA and the Queensland and SWP CA-MRSA clones in Australia have previously been reported [18,31,32]. Patients colonized or infected with the Bengal Bay clone have been observed to be epidemiologically linked to Indian healthcare workers (unpublished data). The USA300, European, Taiwanese and Bengal Bay CA-MRSA clones are not frequently isolated in WA. This may be due, in part, to WA Health Department infection control interventions applied to patients who are colonized or infected with international PVL positive pandemic clones. A seventh pandemic clone has recently been identified. The DNA microarray profile and the SCC*mec *element of the PVL negative ST398-V [5C2&5] is indistinguishable from the pandemic ST398 clone initially isolated from pigs and pig farmers in the Netherlands [39]. Only one isolate, from a patient with travel outside of Australia, has been identified in WA.

The Queensland clone (ST93-IVa [2B]) first detected on the east coast of Australia in the Caucasian population in 2000 [40], has become one of the most prevalent CA-MRSA isolated in Australia [18] and in 2010 accounted for 18% of CA-MRSA in WA. This suggests the acquisition of the SCC*mec *element has given this clone a selective advantage. Although the Queensland clone is believed to have been introduced into WA in 2001 [22], PVL positive ST93-MSSA was identified as the most prevalent *S. aureus *clone in WA's remote indigenous communities in surveys performed in the mid 1990s. Although found in an environment of high β-lactam use a methicillin-resistant variant of ST93-MSSA was not found in WA during these surveys.

WA1, WA2 and WA3 are PVL negative and do not harbor multiple virulence genes (Tables 1). Similarly the successful Queensland clone, although PVL positive, carries almost no other exotoxin genes and no additional resistance genes. Although most other WA CA-MRSA clones are also PVL negative, many of these clones have acquired multiple resistance and/or virulence determinants (Tables 1). For example WA78 (ST188-IVa [2B]/t315) in addition to *mecA *and *blaZ*, harbors *aacA*-*aphD*, *tetK *and *cat *and is phenotypically resistant to erythromycin, trimethoprim and ciprofloxacin; WA64 (ST5-IVa [2B]/t3778) has acquired *seA *enterotoxin genes and *edinA *and *lukF-PV lukS-PV *virulence genes; and WA62 (ST923[ST8slv]-IVa [2B]/t1635) harbors *seD*+*seJ*+*seR *and *seK+seQ *enterotoxin genes and *lukF-PV lukS-PV*. The acquisition of multiple resistance and/or virulence genes may have come at a high fitness cost as none of these clones have established a niche in the WA community.

As WA1, WA2 and WA3 CA-MRSA lack PVL as well as other virulence genes that are found in pandemic international CA-MRSA clones, such ACME in USA300, the epidemiology of CA-MRSA disease in WA is different to other regions. Outside of WA the majority of diseases related to CA-MRSA infection are severe skin and soft tissue infections such as soft tissue abscess, carbuncles and furuncles. Many of these infections have occurred in healthy individuals, especially children and adolescents, usually via skin-to-skin contact [41]. In WA the majority of CA-MRSA related diseases were initially associated with the indigenous population and then other groups normally susceptible to *S. aureus *infections such as the elderly. As the original WA CA-MRSA are PVL negative, many of these infections were superficial skin infections such as impetigo. However with the introduction of the PVL-positive Queensland CA-MRSA clone more severe skin and soft tissues infections have been observed.

The limitation of this study is that only the initial isolate of each PFGE pulsotype was included in the study. To determine if the successful CA-MRSA clones found in the WA community are evolving the genetic profiles of subsequent isolates need to be investigated.

## Conclusions

In conclusion although the vertical and horizontal transmission of SCC*mec *elements into *S. aureus *has occurred on multiple occasions in the WA community only three WA CA-MRSA clones have found an ecological niche. These three PVL negative clones harbor few additional resistance and virulence genes which paradoxically may account for their success.

## Methods

### Isolates

**Figure 3:**
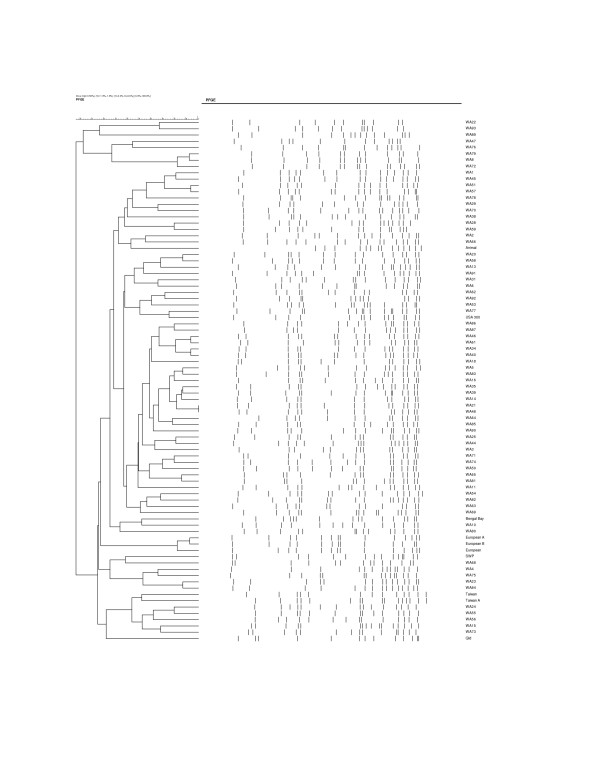
**Dendrogram of the 83 pulsed-field gel electrophoresis patterns of CA-MRSA isolated in Western Australia**.

The isolates studied are representative of the 83 CA-MRSA unique PFGE strains identified in WA from 1989 to 2010 (Figure 3). They include five strains isolated from indigenous inhabitants living in remote WA rural communities in 1989 (WA5 {WBG7583} [20]) and 1995 (WA1 {WBG 8287}, WA2 {WB8366}, WA3 {WBG8378}, and WA4 {WBG8404} [42]); and 78 strains identified from 24,368 CA-MRSA referred to ACCESS Typing and Research between July 2003 and June 2010.

### *nuc *and *mecA*

*S. aureus *species and methicillin resistance was confirmed by the detection of *nuc *(thermostable extracellular nuclease) and *mecA *(methicillin resistance) genes by PCR [43].

### Susceptibility testing

An antibiogram was performed by disk diffusion on Mueller-Hinton agar according to the Clinical and Laboratory Standards Institute (CLSI) recommendations [44]. A panel of eight antimicrobial drugs was tested: erythromycin (15 μg), tetracycline (30 μg), trimethoprim (5 μg), ciprofloxacin (5 μg), gentamicin (10 μg), rifampin (5 μg), fusidic acid (10 μg), and mupirocin (5 μg). CLSI interpretive criteria [45] were used for all drugs except fusidic acid [46] and mupirocin [47].

### PVL

PCR for the detection of PVL determinants was performed as previously described [48].

### PFGE

Electrophoresis of chromosomal DNA was performed as previously described [49], using a contour-clamped homogeneous electric field (CHEF) DR III system (Bio-Rad Laboratories Pty Ltd). Chromosomal patterns were examined visually, scanned with a Quantity One device (Bio-Rad Laboratories Pty Ltd), and digitally analyzed using FPQuest (Bio-Rad Laboratories Pty Ltd). *S. aureus *strain NCTC 8325 was used as a reference strain.

### MLST and *spa *typing

Chromosomal DNA for MLST and *spa *typing was prepared using a DNeasy tissue kit (Qiagen Pty Ltd).

MLST was performed as previously described [50]. The sequences were submitted to http://www.mlst.net/ where an allelic profile was generated and an ST assigned. Clonal complex (CC) was determined using the eBURST V3 algorithm at the same website. Clones that diverged at no more than one of the seven MLST loci were considered to belong to the same CC. Double locus variants (dlvs) were included if the linking single locus variant (slv) was present in the MLST database.

*spa *typing, a DNA sequenced-based analysis of the protein A gene variable region was performed as previously described [51] using the nomenclature as described on the Ridom website (http://spa.ridom.de/).

### SCC*mec *typing

The strategy used for SCC*mec *typing was as previously described [32]. SCC*mec *nomenclature is used as proposed by the International Working Group on the Classification of Staphylococcal Cassette Chromosome Elements (IWG-SCC) [52]. Briefly, the structural type is indicated by a Roman numeral, with a lowercase letter indicating the subtype, and the *ccr *complex and the *mec *complex are indicated by an Arabic numeral and an uppercase letter respectively in parenthesis. Where there is an extra *ccr *element, this is indicated by "&" and an Arabic numeral designating the *ccr *type. When there is an extra *ccr *element present whose precise location is unknown it is indicated by an "&" and *ccr *number outside the parentheses.

### DNA microarray

Arrays and reagents were obtained from Alere Technologies, Jena Germany. The principle of the assay, related procedures, and a list of targets has been described previously [53,54]. An iterated, linear primer elongation was employed for the simultaneous amplification of all targets. An alternative protocol was used for a few isolates in which amplification and labeling was directed by random primers [55]. This method detects target genes for which the binding sites of the primers used in the first protocol were deleted or changed by nucleotide polymorphisms. Target genes included species markers, markers for accessory gene regulator (*agr*) alleles and capsule types, virulence factors, resistance genes, staphylococcal superantigen-like/exotoxin-like genes (*set/ssl *genes) and genes encoding adhesion proteins. Probes for *mecA*, *ugpQ*, *xylR*, and two probes for *mecR *were used for SCC*mec *typing. The last two probes allowed detection and discrimination of untruncated *mecR *and Δ*mecR*, respectively. Probes for the recombinase genes *ccrA1*, *ccrB1*, *ccrA2*, *ccrB2*, *ccrA3*, *ccrB3*, *ccrA4*, *ccrB4*, and *ccrC1; *the fusidc acid resistance marker Q6GD50; and the J region proteins, *dcs*, *pls-*SCC and the *kdp*-operon were also included.

### MRSA Strain Definition

MRSA strains are defined according to their unique PFGE pulsotype

### MRSA Clone Definition

MRSA clones are defined by the combination of the multilocus sequence type (ST) and the SCC*mec *type [56]. For instance ST1-SCC*mec *IVa [2B] is abbreviated as ST1-IVa [2B].

## Authors' contributions

GC designed the study, analysed and interpreted the data, and drafted the manuscript. SM assisted in the analysis and interpretation of data, and critically revised the manuscript for important intellectual content. JP, HL-T, Y-KC and LE carried out the laboratory procedures. RE critically revised the manuscript for important intellectual content. FGO assisted in the design of the study, analysed and interpreted the data, and critically revised the manuscript for important intellectual content. KJC assisted in the design of the study, analysed and interpreted the data, and critically revised the manuscript for important intellectual content. All authors read and approved the final manuscript.

## Supplementary Material

Additional file 1 **Characterisation of CA-MRSA isolated in Western Australia**. Click here for file

Additional file 2 **DNA Microarray Targets, Primers and Probes**. Click here for file
