# Supplementary material for: Evolution and diversity of community-associated methicillin-resistant Staphylococcus aureus in a geographical region
Source: BMC Microbiol. 2011 Sep 29;11:215. doi: 10.1186/1471-2180-11-215 (PMC3197503; doi:10.1186/1471-2180-11-215)
Supplement: Additional file 1 — Characterisation of CA-MRSA isolated in Western Australia. [file 1471-2180-11-215-S1.DOC]

**Additional File 1: Characterisation of CA-MRSA isolated in Western Australia**

| **PFGE**  **Pulsotype** | **Year** | **Reference**  **Number** | **MLST** | ***spa***  **type** | ***agr***  **type** | | **Capsule**  **type** | **SCC*mec***  **type** | **Antibiogram** | **DNA Microarray Resistance**  **Genotype** | ***lukF/S- PVL*** | **Enterotoxins** | **Bacteriophage Associated**  **Virulence Genes** |
| --- | --- | --- | --- | --- | --- | --- | --- | --- | --- | --- | --- | --- | --- |
| **Clonal Complex 1** | | | | | | | | | | | | | |
| **Group 1** | | | | | | | | | | | | | |
| WA1 | 1995 | WBG8287 | 1 | t127 | III | | 8 | IVa [2B] | Ox^R^ Em^R^ FA^R^ | *mecA, blaZ, ermC,* Q6GD50 |  | *seA, seH, seK+seQ* | *sak, scn* |
| WA45 | 2006 | 06-16252 | 872 | t127 | III | | 8 | IVa [2B] | Ox^R^ Em^R^ FA^R^ Tm^R^ Mp^R^ | *mecA, blaZ, ermC,* Q6GD50, (*dfrA), mupR* |  | *seA, seH, seK+seQ* | *sak, scn* |
| WA57 | 2007 | 07-16124 | 1005 | t127 | III | | 8 | IVa [2B] | Ox^R^ Gm^R^ Tm^R^ | *mecA, blaZ, aacA-aphD, dfrA* |  | *seA, seH, seK+seQ* | *sak, scn* |
| **Group 2** | | | | | | | | | | | | | |
| WA10 | 2003 | 03-16918 | 573 | t5073 | II | | 5 | V [5C2] | Ox^R^ Tm^R^ | *mecA, blaZ, fosB* |  | *seC+seL*, *egc*-cluster, ORF CM14 | *scn* |
| **Bengal Bay MRSA** | | | | | | | | | | | | | |
| Bengal Bay | 2007 | 07-17048 | 772 | t3387 | II | | 5 | V [5C2] | Ox^R^ Em^R^ Gm^R^ Tm^R^ Cp^R^ | *mecA, blaZ, msr(A), mpbBM,aacA-aphD, aphA, sat, fosB* | *lukF/S- PVL* | *seA, seC+seL, egc*-cluster, ORF CM14 | *scn* |
| **Clonal Complex 5** | | | | | | | | | | | | | |
| **Group 1** | | | | | | | | | | | | | |
| WA51 | 2007 | 07-15545 | 6 | DNA | I | | 8 | IVa [2B] | Ox^R^ | *mecA, blaZ, fosB* |  | *seA* | *sak, scn* |
| WA66 | 2007 | 07-17366 | 6 | t701 | I | | 8 | IVa [2B]&5 | Ox^R^ | *mecA, blaZ, fosB* |  | *seA*, *seK+seQ* | *sak, scn* |
| **Group 2** | | | | | | | | | | | | | |
| WA3 | 1995 | WBG8378 | 5 | t002 | II | | 5 | IVa [2B] | Ox^R^ Em^R^ | *mecA, blaZ, ermC, fosB,qacC* |  | *seP, egc*-cluster | *sak, chp, scn* |
| WA64 | 2007 | 07-16986 | 5 | t3778 | II | | 5 | IVa [2B] | Ox^R^ | *mecA, blaZ, fosB* | *lukF/S- PVL* | *seA*, *egc*-cluster | *sak, chp, scn* |
| WA71 | 2008 | 08-17330 | 5 | t002 | II | | 5 | IVa [2B] | Ox^R^ | *mecA, blaZ, fosB* |  | *seP, egc*-cluster | *sak, chp, scn* |
| WA82 | 2009 | 09-15628 | 5 | t002 | II | | 5 | IVa [2B] | Ox^R^ Tm^R^ | *mecA, blaZ, dfrA, fosB, qacA, qacC* |  | *seP*, *egc*-cluster | *sak, chp, scn* |
| WA25 | 2004 | 04-15184 | 575 | t002 | II | | 5 | IVa [2B] | Ox^R^ | *mecA, blaZ, fosB* |  | *seP*, *egc*-cluster | *sak, chp, scn* |
| WA50 | 2006 | 06-18615 | 73 | t002 | II | | 5 | IVa [2B] | Ox^R^ | *mecA, blaZ, fosB* |  | *seP*, *egc*-cluster | *sak, chp, scn* |
| WA65 | 2008 | 08-15231 | 73 | t002 | II | | 5 | IVa [2B] | Ox^R^ Gm^R^ Mp^R^ | *mecA, blaZ, aacA-aphD, aadD, mupR, fosB, qacC* |  | *seP*, *egc*-cluster | *sak, chp, scn* |
| WA74 | 2008 | 08-19202 | 5 | t002 | II | | 5 | IVc [2B] | Ox^R^ | *mecA, blaZ, fosB, qacC* |  | *seP, egc*-cluster | *sak, chp, scn* |
| WA39 | 2005 | 05-18015 | 526 | t4065 | II | | 5 | IV [2B] | Ox^R^ Em^R^ FA^R^ | *mecA,* Q6GD50*, fosB* |  | *egc*-cluster | *sak, chp, scn* |
| WA14 | 2003 | 03-17796 | 5 | t442 | II | | 5 | V [5C2] | Ox^R^ Te^R^Tm^R^ FA^R^ | *mecA, tetM,* Q6GD50*, fosB* |  | *seP*, *egc*-cluster | *sak, scn* |
| WA35 | 2005 | 05-16810 | 5 | t688 | II | | 5 | V [5C2] | Ox^R^ Em^R^ Te^R^ FA^R^ Cp^R^ | *mecA, blaZ, ermC, aadD, tetK, tetM, fexA, fosB* |  | *seD+seJ+seR, egc*-cluster | *sak, chp, scn* |
| WA81 | 2009 | 09-16404 | 5 | t045 | II | | 5 | V [5C2] | Ox^R^ Gm^R^ | *mecA, blaZ, fosB, qacC* |  | *seP*, *egc*-cluster | *sak, chp, scn* |
| WA90 | 2009 | 09-20177 | 5 | t1265 | II | | 5 | V [5C2] | Ox^R^ Em^R^ Tm^R^ Cp^R^ | *mecA, blaZ, ermC, fosB* |  | *seP*, *seD, seJ, egc*-cluster | *sak, (scn)* |
| WA11 | 2003 | 03-17833 | 5 | t045 | II | | 5 | V [5C2&5] | Ox^R^ Gm^R^ | *mecA, blaZ, aacA-aphD, fosB, qacA* |  | *seD+seJ+seR, egc*-cluster | *sak, scn* |
| WA86 | 2009 | 09-18986 | 5 | t002 | II | | 5 | V [5C2&5] | Ox^R^ Gm^R^ Tm^R^ | *mecA, aacA-aphD, fosB, qacC* |  | *seP*, *egc*-cluster | *sak, chp, scn* |
| WA34 | 2005 | 05-17463 | 5 | t458 | II | | 5 | V [5C2&5] | Ox^R^ | *mecA, fosB* |  | *seD+seJ+seR*, *egc*-cluster | *sak, chp, scn* |
| WA80 | 2009 | 09-15037 | 5 | t071 | II | | 5 | V [5C2&5] | Ox^R^ | *mecA, fosB* |  | *seD, seJ, egc*-cluster | *sak, chp, scn* |
| WA85 | 2009 | 09-17872 | 5 | t2666 | II | | 5 | V [5C2&5] | Ox^R^ Tm^R^ | *mecA, blaZ, fosB* |  | *seP*, *egc*-cluster | *sak, chp, scn* |
| WA87 | 2009 | 09-18264 | 835 | t002 | II | | 5 | V [5C2&5] | Ox^R^ Cp^R^ | *mecA, blaZ, fosB* |  | *(*seB*), seC+seL, seD+seJ+seR, egc*-cluster | *sak, chp, scn* |
| WA61 | 2007 | 07-18115 | 641 | t002 | II | | 5 | V [5C2]&2 | Ox^R^ Cp^R^ | *mecA, blaZ, fosB, qacC* |  | *seD+seJ+seR*, *egc*-cluster | *sak, scn* |
| WA40 | 2005 | 05-18551 | 835 | t002 | II | | 5 | V [5C2&5]&2 | Ox^R^ Cp^R^ | *mecA, blaZ, fosB, qacC* |  | *seD+seJ+seR, egc*-cluster | *sak, chp, scn* |
| WA46 | 2006 | 06-16677 | 835 | t002 | II | | 5 | V [5C2&5]&2 | Ox^R^ Cp^R^ | *mecA, blaZ, fosB, qacC* |  | *seD+seJ+seR*, *egc*-cluster | *sak, scn* |
| WA18 | 2004 | 04-16891 | 5 | t002 | II | | 5 | novel B | Ox^R^ | *mecA, blaZ, fosB* |  | *seP, seD+seJ+seR, seK+seQ, egc*-cluster | *sak, chp, scn* |
| WA21 | 2004 | 04-17091 | 5 | t002 | II | | 5 | novel B | Ox^R^ | *mecA, blaZ, fosB, qacC* |  | *seA*, *seD+seJ+seR, egc*-cluster | *sak, scn* |
| WA48 | 2006 | 06-17586 | 835 | t002 | II | | 5 | novel B | Ox^R^ Cp^R^ | *mecA, blaZ, fosB* |  | *seD+seJ+seR, egc*-cluster | *sak, chp, scn* |
| **Clonal Complex 8** | | | | | | | | | | | | | |
| WA5 | 1989 | WBG7583 | 8 | t008 | I | | 5 | IVa [2B] | Ox^R^ Te^R^ | *mecA, blaZ, tetK, fosB* |  |  | *sak, chp, scn* |
| WA6 | 2003 | 03-15521 | 8 | t008 | I | | 5 | IVa [2B] | Ox^R^ Te^R^ | *mecA, blaZ, tetK, fosB* |  |  |  |
| WA62 | 2007 | 07-18116 | 923 | t1635 | I | | 5 | IVa [2B] | Ox^R^ Em^R^ Te^R^ | *mecA, blaZ, msr(A), mpbBM, tetK, fosB* | *lukF/S- PVL* | *seD+seJ+seR, seK+seQ* | *sak, chp, scn* |
| WA83 | 2009 | 09-17714 | 1634 | t711 | I | | 5 | IVa [2B] | Ox^R^ Em^R^ Gm^R^ Tm^R^ Rf^R^ | *mecA, blaZ, aacA-aphD, dfrA, fosB* |  |  | *sak, chp, scn* |
| WA58 | 2007 | 07-16233 | 1173 | t064 | I | | 5 | IVd [2B] | Ox^R^ Em^R^ Gm^R^ Te^R^ Tm^R^ Rf^R^ | *mecA, blaZ, ermA, aacA-aphD, aphA, sat, tetM, dfrA, fosB* |  | *seA, seB+seK+seQ* | *sak, scn* |
| WA20 | 2004 | 04-17052 | 612 | t064 | I | | 5 | IVd [2B] | Ox^R^ Gm^R^ Te^R^ Tm^R^ Rf^R^ | *mecA, blaZ, aacA-aphD, tetM, dfrA, cat, fosB* |  | *seA, seB+seK+seQ* | *sak, scn* |
| WA92 | 2010 | 10-15552 | 1757 | t024 | I | | 5 | IVa [2B]&5 | Ox^R^ Tm^R^ | *mecA, blaZ, dfrA, fosB* |  |  | *scn* |
| WA31 | 2005 | 05-15529 | 576 | t334 | I | | 5 | IV [2B] | Ox^R^ | *mecA, blaZ, fosB* |  |  |  |
| WA77 | 2008 | 08-20001 | 8 | t008 | I | | 5 | V [5C2] | Ox^R^ Te^R^ FA^R^ | *mecA, blaZ, tetK,* Q6GD50*, fosB, qacC* |  | *seA, seK+seQ* | *sak, scn* |
| WA53 | 2006 | 06-18088 | 8 | t2238 | I | | 5 | V [5C2&5] | Ox^R^ Em^R^ Gm^R^ Cp^R^ | *mecA, blaZ, ermC, aacA-aphD, fosB* |  | *seA, seB+seK+seQ* | *sak, scn* |
| WA16 | 2003 | 03-16758 | 8 | t024 | I | | 5 | VIII [4A] | Ox^R^ Em^R^ Gm^R^ Cp^R^ Mp^R^ | *mecA, blaZ, ermA, aacA-aphD, mupR, fosB, qacC* |  | *seA* | *sak, scn* |
| **USA300 MRSA** | | | | | | | | | | | | | |
| USA300 | 2004 | 04-15086 | 8 | t008 | I | | 5 | IVc [2B] | Ox^R^ Te^R^ | *mecA, blaZ, tetK, fosB* | *lukF/S- PVL* | *seK+seQ* | *sak, chp, scn* |
| **Clonal Complex 9** | | | | | | | | | | | | | |
| WA13 | 2003 | 03-17992 | 834 | t3029 | I | | 8 | IVc [2B] | Ox^R^ Em^R^ | *mecA, blaZ, msr(A), fosB* |  | *seC+seL* | *sak, chp, scn* |
| **Clonal Complex 12** | | | | | | | | | | | | | |
| WA69 | 2007 | 07-19013 | 12 | t160 | II | | 8 | IVa [2B] | Ox^R^ | *mecA, blaZ, fosB* |  | *seP, seB*, ORF CM14 | *sak, scn* |
| WA59 | 2007 | 07-16590 | 12 | t160 | II | | 8 | novel A | Ox^R^ | *mecA, fosB* |  | *seP, seB*, ORF CM14 | *sak, scn* |
| **Clonal Complex 30** | | | | | | | | | | | | | |
| WA68 | 2008 | 08-15775 | 39 | t2643 | III | | 8 | IVc [2B] | Ox^R^ | *mecA, blaZ, fosB* |  | *seA, seC+seL, seO* | sak, scn |
| **South Western Pacific MRSA** | | | | | | | | | | | | | |
| SWP | 2002 | 02-16663 | 30 | t019 | III | | 8 | IVc [2B] | Ox^R^ | *mecA, blaZ, fosB* | *lukF/S- PVL* | *egc*-cluster | sak, chp, scn |
| **Clonal Complex 45** | | | | | | | | | | | | | |
| **Group 1** | | | | | | | | | | | | | |
| WA75 | 2003 | 03-17163 | 45 | t1424 | I | | 8 | IVa [2B] | Ox^R^ | *mecA, blaZ* |  | *(seB), seC+seL, egc*-cluster | *sak, chp, scn* |
| WA4 | 1995 | WBG8404 | 45 | t123 | I | | 8 | V [5C2] | Ox^R^ | *mecA, blaZ* |  | *seK+seQ, egc*-cluster | *sak, chp, scn* |
| **Group 2** | | | | | | | | | | | | | |
| WA23 | 2004 | 04-16679 | 45 | t1575 | IV | | 8 | IVc [2B] | Ox^R^ | *mecA, blaZ* |  | *seJ+ seR*, *egc*-cluster | *sak, chp, scn* |
| WA84 | 2007 | 07-16502 | 45 | t1081 | IV | | 8 | V [5C2&5] | Cp^R^ | *mecA, blaZ* |  | *seJ,* *egc*-cluster | *sak, chp, scn* |
| **Clonal Complex 59** | | | | | | | | | | | | | |
| WA55 | 2007 | 07-15432 | 59 | t437 | I | | 8 | IVa [2B] | Em^R^ Te^R^ | *mecA, blaZ, aphA, sat, tetK* | *lukF/S- PVL* | *seB+seK+seQ* | *sak, chp, scn* |
| WA56 | 2007 | 07-15443 | 59 | t437 | I | | 8 | IVa [2B] | Em^R^ | *mecA, blaZ, aphA, sat, cat* | *lukF/S- PVL* | *seA, seB+seK+seQ* | *sak, chp, scn* |
| WA73 | 2005 | 05-16512 | 59 | t528 | I | | 8 | IVb [2B] | Ox^R^ | *mecA, blaZ* |  | *seB+seK+seQ* | *chp, scn* |
| WA24 | 2004 | 04-17626 | 87 | t216 | I | | 8 | IVb [2B] | Em^R^ | *mecA, blaZ, msr(A), mpbBM, aphA, sat* |  | *seB+seK+seQ* | *sak, chp, scn* |
| WA15 | 2003 | 03-17565 | 59 | t976 | I | 8 | | IVa [2B]&5 | Ox^R^ | *mecA, blaZ* |  | *seA, seB+seK+seQ* | *sak, chp, scn* |
| **Taiwan CA-MRSA** | | | | | | | | | | | | | |
| Taiwan | 2003 | 03-16672 | 59 | t437 | I | 8 | | V [5C2&5] | Em^R^ Te^R^ | *mecA, blaZ, aphA, sat, tetK, cat* | *lukF/S- PVL* | *seB+seK+seQ* | *chp, scn* |
| Taiwan^A^ | 2007 | 07-15076 | 952 | t1950 | I | 8 | | V [5C2&5] | Em^R^ | *mecA, blaZ, aphA, sat, cat* | *lukF/S- PVL* | *seB+seK+seQ* | *chp, scn* |
| **Clonal Complex 72** | | | | | | | | | | | | | |
| WA44 | 2006 | 06-15803 | 72 | t791 | I | | 5 | IVa [2B] | Ox^R^ Tm^R^ | *mecA, blaZ, dfrA, fosB* | *lukF/S- PVL* | *seC+seL, egc*-cluster | *sak, chp, scn* |
| WA91 | 2010 | 10-15302 | 72 | t3092 | I | | 5 | V [5C2] | Ox^R^ Gm^R^ Tm^R^ Cp^R^ | *mecA, blaZ, aacA-aphD, fosB* |  | *seC+seL, egc*-cluster | *sak,scn* |
| **Clonal Complex 75** | | | | | | | | | | | | | |
| WA8 | 2003 | 03-17848 | 75 | ND | NT | | NT | IVa (2B] | Ox^R^ | *mecA, blaZ, fosB, qacC* |  | *seB, egc*-cluster | *sak,scn* |
| WA79 | 2008 | 08-18362 | 75 | ND | NT | | NT | IVa [2B] | Ox^R^ | *mecA, blaZ, fosB* |  | *egc*-cluster | *sak,scn* |
| WA72 | 2008 | 08-16706 | 1304 | ND | NT | | NT | IVa [2B] | Ox^R^ Em^R^ Mp^R^ | *mecA, blaZ, ermC, aadD, mupR, fosB* |  | *seD+seJ+seR*, *egc*-cluster | *sak,scn* |
| **Clonal Complex 80** | | | | | | | | | | | | | |
| **European CA-MRSA** | | | | | | | | | | | | | |
| European | 2005 | 05-17006 | 80 | t044 | III | | 8 | IVc [2B] | Ox^R^ Te^R^ FA^R^ | *mecA, blaZ, aphA, sat, tetK, far1* | *lukF/S- PVL* |  | *sak, scn* |
| European^A^ | 2004 | 04-15395 | 583 | t044 | III | | 8 | IVc [2B] | Ox^R^ Te^R^ FA^R^ | *mecA, blaZ, aphA, sat, tetK, far1* | *lukF/S- PVL* |  | *sak, scn* |
| European^B^ | 2005 | 05-15062 | 728 | t044 | III | | 8 | IVc [2B] | Ox^R^ | *mecA, aphA, sat* | *lukF/S- PVL* |  | *sak, scn* |
| **Clonal Complex 88** | | | | | | | | | | | | | |
| WA2 | 1995 | WBG8366 | 78 | t3205 | III | | 8 | IVa [2B] | *Ox^R^, Em^R^* | *mecA, blaZ, ermA* |  | *seC+seL* | *sak, chp, scn* |
| **Clonal Complex 97** | | | | | | | | | | | | | |
| WA54 | 2007 | 07-15754 | 953 | t359 | I | | 5 | IVa [2B] | Ox^R^ |  |  |  | sak, scn |
| WA63 | 2007 | 07-17920 | 1174 | t267 | I | | 5 | IVa [2B] | Ox^R^ |  |  |  | sak, scn |
| **Clonal Complex 121** | | | | | | | | | | | | | |
| WA22 | 2004 | 04-16237 | 577 | t3025 | IV | | 8 | V [5C2] | Ox^R^ Em^R^ | *mecA, blaZ, ermA, fosB* |  | *egc*-cluster, ORF CM14 | *sak, scn* |
| WA93 | 2010 | 10-15882 | 121 | t159 | IV | | 8 | V [5C2&5] |  | *mecA, blaZ, fosB* |  | *seB, egc-*cluster, ORF CM14 | *sak, scn* |
| **Clonal Complex 152** | | | | | | | | | | | | | |
| WA89 | 2009 | 09-20065 | 1633 | t355 | I | | 5 | V [5C2] | Ox^R^ Te^R^ Tm^R^ | *mecA, blaZ, teK* | *lukF/S- PVL* |  | *sak, scn* |
| **Clonal Complex 188** | | | | | | | | | | | | | |
| WA38 | 2005 | 05-17762 | 188 | t189 | I | | 8 | IVa [2B] | Ox^R^ Em^R^ Gm^R^ Tm^R^ Cp^R^ Rf^R^ | *mecA, blaZ, aacA-aphD* |  |  | *sak, chp, scn* |
| WA78 | 2008 | 08-20097 | 188 | t189 | I | | 8 | IVa [2B] | Ox^R^ Em^R^ Gm^R^ Te^R^ Tm^R^ Cp^R^ | *mecA, blaZ, aacA-aphD, tetK, cat* |  |  | *sak, chp, scn* |
| **Clonal Complex 361** | | | | | | | | | | | | | |
| WA29 | 2005 | 05-15441 | 672 | t1309 | I | | 8 | IVa [2B] | Ox^R^ Cp^R^ | *mecA, blaZ, fosB* |  | *seB, egc-*cluster | *sak, scn* |
| WA70 | 2008 | 08-18855 | 672 | t1309 | I | | 8 | V [5C2] | Ox^R^ Gm^R^ Tm^R^ Cp^R^ | *mecA, blaZ, aacA-aphD, aphA, sat, fosB* |  | *egc-*cluster |  |
| WA28 | 2005 | 05-16157 | 361 | t315 | I | | 8 | VIII [4A] | Ox^R^ Te^R^ | *mecA, blaZ, aphA, sat, tetK, fosB* |  | *egc*-cluster | *sak, chp, scn* |
| **Clonal Complex 398** | | | | | | | | | | | | | |
| Animal | 2009 | 09-16670 | 398 | t034 | I | | 5 | V [5C2&5] | Ox^R^ Em^R^ Te^R^ Tm^R^ | *mecA, blaZ, ermA,* (*tetK*)*, tetM* |  |  |  |
| **Singletons** | | | | | | | | | | | | | |
| WA47 | 2006 | 06-16607 | 883 | t7462 | NT | NT | | IVd [2B] | Ox^R^ Em^R^ | *mecA, ermC, (cat)* |  | *seB* |  |
| Queensland CA-MRSA | | | | | | | | | | | | | |
| Qld | 2003 | 03-16790 | 93 | t202 | III | 8 | | IVa [2B] | Ox^R^ | *mecA, blaZ* | *lukF/S- PVL* | ORF CM14 | *sak, chp, scn* |
| **Clonal Complex not Determined** | | | | | | | | | | | | | |
| WA76 | 2006 | 06-17540 | 1303 | ND | III | | NT | IVa [2B] | Ox^R^ | *mecA, blaZ, fosB* |  | *seB, egc-*cluster | *scn* |

PFGE, pulsed field gel electrophoresis; MLST, multilocus sequence type; *spa* Type: ND, Not Determined; NT, Non typeable; *agr*, accessory gene regulator

SCC*mec*, staphylococcal cassette chromosome *mec*;

Antibiogram: Ox, oxacillin; Cp, ciprofloxacin; Em, erythromycin; FA, fusidic acid; Gm, gentamicin; Mp, mupirocin; Rf, rifampicin; Te, tetracycline

Resistance Genotype: *mecA*, methicilin; *aacA-aphD*, aminoglycoside; *aadD*, tobramycin; *aphA*, neomycin/kanamycin *blaZ*, beta lactamase; *cat*, chloramphenicol; *dfrA*, trimethoprim; *ermA*, erythromycin/clindamycin; *ermC*, erythromycin/clindamycin; *far1*, fusidic acid; *fosB*, fosfomycin; *mpbBM*, lysylphosphatidylglycerol synthetase; *msr[a]*, mercuric; *mupR*, mupirocin; Q6GD50, fusidic acid; *qacA* quaternary ammonium compound; *qacC* quaternary ammonium compound; *sat*, streptomycin; *tetM*, *tetracycline*; tetK, tetracycline;
